# Machine Learning Assists IoT Localization: A Review of Current Challenges and Future Trends

**DOI:** 10.3390/s23073551

**Published:** 2023-03-28

**Authors:** Reza Shahbazian, Giusy Macrina, Edoardo Scalzo, Francesca Guerriero

**Affiliations:** Department of Mechanical, Energy and Management Engineering (DIMEG), University of Calabria, 87036 Rende, Italy

**Keywords:** machine learning, localization, Internet of things, fingerprinting, Industry 4.0

## Abstract

The widespread use of the internet and the exponential growth in small hardware diversity enable the development of Internet of things (IoT)-based localization systems. We review machine-learning-based approaches for IoT localization systems in this paper. Because of their high prediction accuracy, machine learning methods are now being used to solve localization problems. The paper’s main goal is to provide a review of how learning algorithms are used to solve IoT localization problems, as well as to address current challenges. We examine the existing literature for published papers released between 2020 and 2022. These studies are classified according to several criteria, including their learning algorithm, chosen environment, specific covered IoT protocol, and measurement technique. We also discuss the potential applications of learning algorithms in IoT localization, as well as future trends.

## 1. Introduction

The term Internet of things (IoT) is used to indicate a network of physical objects, including machinery, furniture, and construction materials, equipped with sensors, software, and connectivity to gather and exchange data [1]. The Internet of things is important because it allows individuals and companies to better comprehend and exert control over their environment, enhancing efficiency, safety, and convenience. The Fourth Industrial Revolution (Industry 4.0) [2] is the term used to describe the present trend of automation and data interchange in manufacturing technology, including the use of the IoT. By utilizing cutting-edge technologies such as artificial intelligence, machine learning, and the Internet of things, Industry 4.0 is crucial because it enables organizations to boost productivity, reduce costs, and improve goods and services. In general, the integration of the IoT and Industry 4.0 is promoting the growth of a society that is more interconnected, intelligent, and productive [3,4].

Localization is the process of pinpointing the precise location of an IoT device. The problem is known as IoT network localization if the goal is to localize all the devices. The utilization of numerous technologies, including GPS, wireless communication signals, and sensor data, can be used to do this [5]. In the context of IoT localization, machine learning can be used to analyze data from many sources [3] to enhance the precision and dependability of position predictions. For instance, trends in data gathered from IoT devices could be examined by machine learning algorithms in order to identify elements that influence the precision of the localization. This may involve the strength of the wireless signal, the presence of objects that obstruct or skew the signals, or the existence of other interference sources. By learning to recognize and account for these characteristics, machine learning algorithms can increase the precision of the localization.

Many IoT localization systems use learning algorithms, which allow the system to adapt and improve its performance over time. IoT localization can be categorized in several ways, such as based on measurement methods, range-based localization, and range-free localization, which can be carried out in both indoor and outdoor settings. Range-based IoT localization refers to methods that leverage an IoT device’s distance from one or more reference points to pinpoint its location [6]. They could be specialized equipment deployed particularly for localization, or they could be other devices fixed in recognized locations such as cell towers or WiFi routers. Range-based IoT localization methods include triangulation, trilateration, and multilateration. Techniques for locating an IoT device without using distance measurements are referred to as range-free IoT localization. Instead, these methods rely on other kinds of data, such as the intensity of the wireless signal, the existence of certain landmarks or other objects, or patterns in the data from the device [7].

The ideal IoT localization method relies on the particular needs of the application. Both range-based and range-free strategies have benefits and drawbacks. Although range-based methods are typically more precise, they could be limited by the lack of reference locations or the precision of distance measurements. Range-free methods may be less precise, but in some circumstances, they are more flexible and simpler to use.

Indoor IoT localization is the process of locating an IoT device inside a structure or another enclosed place. Because the gadget could not have access to GPS or other external signals for localization, this can be a challenging process. Instead, indoor localization systems usually rely on other kinds of information, like the intensity of the wireless signal, the existence of particular landmarks or other objects, or patterns in the data that the device collects. Outdoor IoT localization is the procedure used to pinpoint an IoT device’s position in an outdoor setting. Because the gadget can commonly use GPS or other exterior signals for localization, this is typically simpler than indoor localization. On the other hand, obstructions or other sources of interference may pose problems for outdoor localization systems, affecting the precision of the position estimates [8,9].

In IoT localization, a variety of wireless radiofrequency techniques could be used. Wireless technologies are critical in an IoT ecosystem because they enable device-to-device communication and data transfer. WiFi, for example, communicates between devices using radio waves, and signal strength can be measured using the received signal strength indicator (RSSI) and channel state information (CSI), which can be used for indoor localization. Bluetooth Low Energy (BLE) is a low-power wireless technology that is used for short-range communication between devices and is also used in IoT localization via beacons. RFID uses radio waves to transfer data between a reader and a tag attached to an object, allowing it to be identified and located. Ultrawideband (UWB) technology transmits data over a wide bandwidth using high-frequency radio pulses, providing fast and accurate location information. Ultrasonic technology, which uses high-frequency sound waves to measure distance and detect objects, is also widely used in IoT localization applications. Some of the most common technologies referred in the covered publications are as follows:

- The received signal strength (RSS) is a measurement of the strength of a wireless signal at a specific location. It is commonly used in IoT localization systems to determine the distance between a device and a reference point, such as a WiFi router or cell tower. By measuring the RSS at multiple reference points, the device’s location can be triangulated. The RSS is typically expressed in decibels relative to one milliwatt. It is a measurement of the strength of a wireless signal at a specific location that can be used to determine the distance between the device and the reference point. The stronger the signal, the shorter the distance. The relationship between the RSS and distance, on the other hand, is not always straightforward, as it can be influenced by a variety of factors such as the presence of obstacles, interference from other signals, and antenna characteristics [10,11].
- The channel state information (CSI) is a measure of the characteristics of a wireless signal, such as its phase and amplitude at different frequencies. It is frequently used in WiFi localization systems to improve the accuracy of location estimates. The CSI can be used to collect data about the environment in which the signal is being transmitted, such as the presence of reflections or specific objects. The CSI is typically expressed in complex numbers that represent the phase and amplitude of the signal at each frequency. The distance between the device and the reference point, the presence of obstacles or other sources of interference, and the antenna characteristics of the device can all influence the signal’s phase and amplitude [12].
- Bluetooth Low Energy (BLE) is a wireless communication technology popular in IoT devices. Because of its low power consumption and short range, it is well-suited for use in location-based services. BLE can be used in both range-based and range-free localization systems, depending on how it is implemented [13].
- Other radiofrequency techniques used in IoT localization include radiofrequency identification (RFID) and ultrawideband (UWB). RFID employs passive tags that are attached to objects and read by a reader, whereas UWB employs radio pulses that are extremely brief in duration and can be used for high-precision localization [14].

There are numerous machine learning algorithms that can be used in IoT localization, some of the common algorithms are presented in the following:

- k-Nearest neighbors (KNN): A simple but effective machine learning algorithm that can be used for both classification and regression. In the context of IoT localization, KNN could be used to predict the location of an IoT device based on the locations of other nearby devices.
- Decision trees: A machine learning algorithm that produces a treelike model of decisions and their potential outcomes. It can forecast an IoT device’s location based on the values of various features, such as the wireless signal strength or the presence of specific landmarks.
- Support vector machine (SVM): SVMs are powerful machine learning algorithms that can be used for classification, regression, and a variety of other tasks. In the context of IoT localization, SVMs could be used to predict the location of an IoT device based on patterns in data collected from it.
- Neural networks are a type of machine learning algorithm that is inspired by the structure and function of the human brain. Neural networks can perform a variety of tasks, including classification, regression, and pattern recognition. In the context of IoT localization, neural networks could be used to predict the location of an IoT device based on patterns in data collected from it.
- Deep learning (DL): DL is a type of machine learning that makes use of deep neural networks with multiple layers of processing. It is capable of detecting complex patterns in data and predicting outcomes based on those patterns. In the context of IoT localization, deep learning could be used to predict the location of an IoT device based on patterns in data collected from it. Deep learning is a subset of machine learning that makes use of deep neural networks with multiple processing layers. Deep learning can be used in IoT localization to analyze data from various sources, such as GPS, wireless communication signals, and sensor data, in order to improve the accuracy and reliability of location estimates. The ability of deep learning to handle large and complex datasets is a significant advantage for IoT localization. Deep learning algorithms can learn to see patterns and relationships in data that humans cannot, allowing them to make more accurate predictions. Deep learning algorithms can also learn and improve their performance as they are exposed to more data.
- Reinforcement learning is a subset of machine learning in which an agent learns how to interact with its surroundings in order to maximize a reward signal. Reinforcement learning could be used in the context of IoT localization to optimize the behavior of an IoT device in order to improve the accuracy of its location estimates.

**Figure 1:**
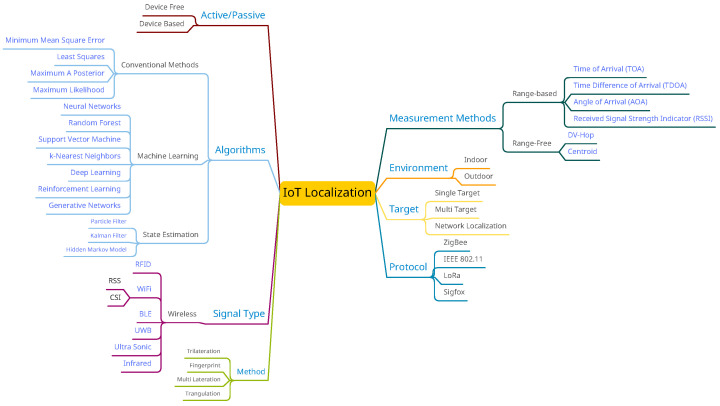
A high-level overview of the IoT localization classification.

Figure 1 depicts a high-level overview of the IoT localization classification.

### 1.1. Our Methodology

**Figure 2:**
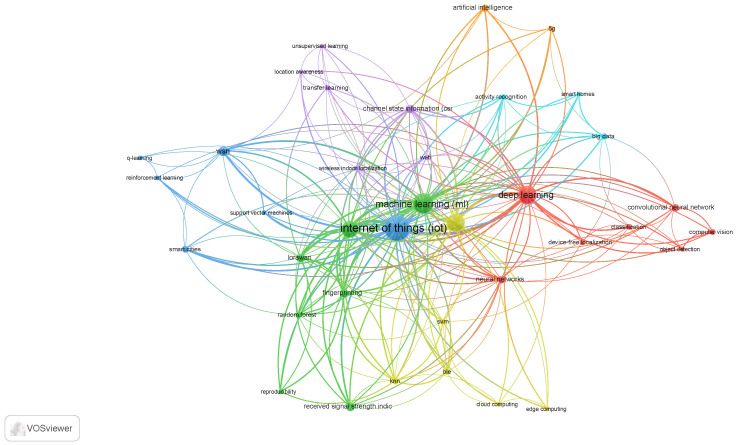
The keyword diagram on the Scopus database search results. This diagram depicts the related keywords used by authors in their papers. In Scopus, we used the “TITLE-ABS-KEY (iot OR (internet AND of AND things) AND localization AND learning)” query, as well as a prepossessing to ensure that similar keywords formed only one cluster.

In this paper, we conducted a search of the Scopus database for publications that included learning, IoT, and were related to localization. Following the completion of the search and collection of papers, the database results were filtered based on title and abstract, followed by a full-text examination of the selected papers. Our primary goal was to cover all related papers that addressed machine learning methods in IoT localization. We covered studies published between 2020 and 2022. Our Scopus database searches included (“IoT” or “Internet of Things”) and (“Localization”) and (“Learning”). We used the Scopus query, “TITLE-ABS-KEY (iot OR ( internet AND of AND things ) AND localization AND learning )” for the search and the VOSviewer application for visualization [15]. The general keyword analysis of all related keywords is shown in Figure 2, which depicts the keywords used by the authors. The related keywords are organized into clusters, each given a different color. In the visualization, the distance between two clusters approximates the relatedness of those keywords.

In the present survey paper we did not employ specific methods to assess the risk of bias in the included studies, as our primary objective was to present a descriptive overview of the available literature sourced from the Scopus database. In this paper, we used a narrative synthesis by presenting the summary of the included studies. We also used the tabular summary to group the studies based on similarities in their methodologies, learning algorithms, and measurement techniques.

### 1.2. Existing Surveys

Asaad and Maghdid wrote one of the most recent and comprehensive surveys in the literature [9]. The authors classified outdoor and indoor localization based on wireless techniques, sensors, environments, objects, and metrics in their paper. The paper spanned the years 2019 to 2021.

Moradbeikie et al. investigated GNSS-free outdoor localization for the IoT with publications published between 2018 and August 2021 in another paper [16]. The authors addressed cloud-, fog-, and edge-AI-based architectures in the paper, with a focus on GNSS-free IoT localization methods. They addressed some limited deep learning or deep neural network (DNN) methods used to improve localization accuracy.

Kordi and colleagues examined wireless emerging IoT indoor localization [17]. They classified the papers based on their localization techniques and discussed some of the learning-based methods used to optimize wireless techniques. The review was published at a conference and covered a small number of publications published between 2016 and 2019.

Rathin Chandra Shit et al. examined prior-year publications on IoT localization and presented a taxonomy of localization methods [5]. The self-determining methods and training-dependent methods were included in the taxonomy. Fingerprinting, a stochastic model process, and a machine learning approach comprised the training models.

Li and colleagues investigated location-enabled IoT and addressed positioning techniques, error sources, and error mitigation [18]. The paper focused on LPWAN technologies such as LTE-M, Sigma, NB-IoT, LoRaWan, Weightless, UNB, and PRMA.

Atitallah et al. investigated the use of deep learning and big data analytics to aid in the development of smart cities [19]. They addressed learning-based localization in passing, as they addressed two publications in the literature published in 2017 and 2018.

Machine learning in WiFi-based indoor positioning was reviewed by Bellavista-Parent, Torres-Sospedra, and Pérez-Navarro [20]. The authors conducted a literature review and categorized existing methods based on machine learning techniques such as deep reinforcement learning (DRL), extreme learning machine (ELM), convolutional neural networks (CNNs), deep neural networks (DNNs), backpropagation neural networks (BPNNs), capsule neural networks (CapsNets), stacked denoising autoencoders (SDAs), variational autoencoder (VAEs), and deep belief networks (DQNs). The survey also covered other methods such as support vector machines (SVMs) and Bayesian methods. The authors covered papers published between 2016 and 2021.

Non-RF techniques for unobtrusive indoor positioning were discussed by Alam, Faulkner, and Parr [21]. The location-based services were addressed using visible light, infrared, vibration, pressure, and an electric field. The studies covered the years 2010 to 2020.

Khan et al. presented a review of location-aware IoT schemes [22]. The papers were classified by the authors based on their applications in an indoor or outdoor environment, smartphone usage, security, energy efficiency, target recovery, and target prediction capabilities. The authors covered publications that were published prior to 2021.

Farahsari and colleagues examined indoor positioning systems for IoT-based applications [23]. The reviewed studies were classified by their scale (local or global), environment (indoor or outdoor), and initial user (tracking or navigation). Time-based, signal-based, and direction-based algorithms were among those examined in the survey. The authors also discussed communication technologies, such as short-range, long-range, and signal-type communication. The survey included studies published prior to 2022 that addressed applications, vendors, and services but did not include any machine learning algorithms.

Mahmood et al. presented a review of machine learning algorithm applications for future IoT toward the 6G era [24]. The survey focused on the evaluation of communication networks (3G to 6G) and categorized the studies covered based on the algorithm type, which included heuristic, supervised, unsupervised, deep learning, reinforcement learning, deep reinforcement learning, and federated learning. They also addressed future open research directions in channel modeling, resource and data management, energy efficiency, security, and privacy based on 6G communication.

Deep learning methods for fingerprint-based indoor positioning were reviewed by Alhomayani and Mahoor [25]. The authors only looked at deep learning methods in a specific type of localization and only looked at studies published before 2020.

Table 1 shows a summary of the existing surveys. There is no specific survey focusing on machine learning techniques and applications in IoT-based localization, as shown in this table.

### 1.3. Contributions

Currently, there are only a few surveys that address machine learning and artificial intelligence algorithms for localization. There are no publications from late 2021 or 2022 in the related surveys, and there is no specific survey focusing on machine learning applications in IoT-based localization. This prompted us to conduct this investigation.

We focus on machine learning algorithms in IoT localization in this paper. We begin by reviewing the relevant literature. These studies are classified based on some extracted features. We discuss some of the current and future applications of IoT localization, followed by the use of learning algorithms to improve their accuracy and reliability. We address some of the issues that arise when using machine learning to localize IoT devices.

In summary, the following are the paper’s contributions:

- By focusing on machine learning algorithms for IoT localization, we fill a gap in existing surveys. We cover publications released between 2020 and 2022.
- We categorize the current literature based on measurement techniques, specific IoT protocols, whether they are range-based or range-free, and the machine learning algorithm that they use.
- We examine the current and potential applications of machine-learning-based IoT localization.
- We discuss the challenges and future trends of machine-learning-based IoT localization.

The remainder of this paper is organized as follows: Section 2 reviews the literature and discusses related works. Section 3 presents the applications of learning algorithms in IoT localization, and the current challenges of learning-based IoT localization are presented in Section 4. The lesson learned, evaluation setup, and future trends are presented in Section 5, and finally, Section 6 concludes the paper.

## 2. Literature Review

We provide a review of the selected publications in this section. We refer to related works based on their publication date. We begin with those published in 2020, then move on to those published in 2021 and 2022. Table 2 summarizes the reviewed literature.

Janssen, Berkvens, and Weyn examined the low-power, wide-area networking (LoRaWAN) benchmarking when RSS-based localization algorithms were used. LoRaWAN is a protocol built on top of the LoRa radio modulation technique that connects devices to the Internet and network gateways wirelessly. The authors compared the accuracy and computational performance of RSS fingerprint-based and range-based location estimation algorithms using a publicly available outdoor LoRaWAN dataset. Their evaluations showed that a trade-off between accuracy and implementation cost was required. The fingerprint-based methods were more accurate, but they required model training, whereas the range-based algorithm could be deployed instantly on any network coverage. They evaluated linear regression, SVM, KNN, and random forest algorithms for fingerprint localization, and the Min-Max and E-Min-Max algorithms for range-based localization [26,27].

Krupanek and Bogacz used artificial neural networks (ANNs) to investigate the localization of IoT nodes. They proposed a two-stage algorithm to improve the localization of RSS-based accuracy. They used RSSI coefficient samples to run the localization algorithm. Their algorithm took into account some sensors with known coordinates, referred to as anchor nodes. To perform the neural network’s learning process, they formed a matrix of RSSI coefficients. The unknown positions of the nodes were fed into the neural network inputs. The authors employed Levenberg–Marquardt supervised learning, Bayesian regularization, and backpropagation. They evaluated various ANN structures that were tested with varying numbers of hidden layers and nodes. The authors demonstrated that the main advantage of their ANN-based method was that it did not require prior knowledge of the environment or noise distribution. RSSI measurements are typically highly unstable and are affected by environmental noise as well as the possibility of moving sensor nodes [28].

An et al. proposed a deep tracking platform for the Internet of things. Their proposed platform was middleware-free and supported NB-IoT, LoRa, RFID, Sigfox, and Zigbee. They proposed a deep tracking framework and used convolutional neural networks (CNNs) to improve tracking accuracy and stability [29].

Bhatti et al. used the RSS to investigate outlier detection for localization in indoor environments. They used the iForest method for unsupervised learning and SVM, KNN, and random forest for supervised learning. In fact, the authors used ensemble learning, which means they combined the methods mentioned above into one for better results. They assessed the proposed method’s performance on publicly available datasets [30].

Sun et al. investigated device-free indoor ZigBee localization and proposed a deep learning convolutional neural network (CNN) model [31]. Yang and Wu used deep neural networks and wireless radio links in the IoT to build a network based on the ZigBee protocol that could be used for single target localization in another study of device-free localization [32].

Wang et al. proposed a three-step framework for robust localization in N-Los environments. They used a hierarchical clustering method to divide the network into several subclusters with a small number of nodes, followed by outlier detection and low-rank matrix completion algorithms to complete the Euclidean distance matrix (EDM), and multidimensional scaling (MDS) to calculate the relative coordinates. Finally, the relative coordinates of all subclusters were transformed for network localization [33].

Deep learning techniques for IoT localization were implemented in 5G networks by Boudani et al. The authors proposed DELTA, a deep-learning-based cooperative architecture built on a 3D multilayered fingerprint radio map based on the RSS. The authors first estimated a 2D location, and the output was recursively used to predict a mobile station’s 3D location [34].

D’Aloia et al. investigated BLE fingerprinting indoor localization using RSS signals. They used KNN and ANN to localize a dataset collected by five anchors in a building [35].

WAN and colleagues investigated machine learning applications for IoT-based vehicular localization. Their system architecture was made up of multiple IoT devices with an arbitrary array configurations and a network of smart-city vehicles. They considered noncircular signals and used the DOA estimation method to extend the vehicle number estimation method to mixed signals. They employed unsupervised learning [36].

Ghorpade, Zennaro, and Chaudhari concentrated on range-based elderly localization in indoor environments using IoT. They proposed a hybrid optimized fuzzy threshold ELM (HOFTELM) algorithm by combining extreme learning machine (ELM), fuzzy system, and modified swarm intelligence. They also employed particle-swarm gray-wolf optimization to determine the motion of the sensor node [37].

Dou et al. investigated RSS-based multifloor fingerprint localization. They modeled the problem as a Markov decision process (MDP) and used deep reinforcement learning to solve it. In particular, the authors employed Q-Learning, which detects the location of a target by successively bisecting the search space to a small cube. This has the potential to reduce the search space and computational complexity [38].

Jia et al. proposed an RSS-based deep neural network for indoor fingerprint localization [39]. The authors of [40] proposed an outdoor localization scheme for LoRaWANs using semisupervised transfer learning. They used the concept of segmentation to generate a large quantity of virtual labeled data. The labeled–unlabeled data relationship was fine-tuned on a regular basis. The accuracy improved as the number of virtual labeled data increased. The main signal features used by the authors were RSSI, SNR, and timestamps.

Kim et al. investigated IoT network localization and proposed a low-rank matrix completion method based on deep learning. They recovered the desired matrix by utilizing the properties of a Euclidean distance matrix, such as its low-rank, symmetric, zero diagonal, and positive nondiagonal entries. To recover the matrix in IoT environments, they expressed it as a function of sensor coordinates and used a deep neural network [41].

Varma and Anand proposed a random-forest-based learning algorithm that focused on improving indoor localization accuracy as an IoT service with a focus on smart buildings. They took into account the area with 13 iBeacons that generated RSSI values [42].

Thakur and Han investigated indoor localization using Bluetooth Low Energy. They detected a user’s location by utilizing multimodal components of user interactions. They also examined some learning methods, such as a random forest, artificial neural network, decision tree, support vector machine, k-NN, gradient boosted trees, deep learning, and linear regression, in order to address the challenge of determining the best machine learning approach for indoor Localization [43].

Tiwary and colleagues investigated deep-learning-based fingerprint localization. They addressed the heterogeneity and temporal variation in RSS values in IoT networks and proposed *r*-vectors as a device-invariant signature of a specific location [44].

Jain et al. investigated low-cost and low-energy solutions for IoT localization. They considered Bluetooth Low Energy (BLE) technology for indoor localization and employed an RSSI-based fingerprinting technique. They used a random forest as a learning technique and compared its accuracy to that of KNN, SVM, and decision trees [45].

Spyridis et al. investigated mobile IoT device tracking in 6G using a group of UAVs outfitted with RSSI sensors. They used a deep learning model based on a graph convolutional network (GCN) architecture to cluster the UAV network at regular intervals. They presumed that the location of the UAVs was known ahead of time (anchor). The number of clusters at any given time was determined by a heuristic method (based on their distance from the source emitting radiofrequency signals), and partitions were determined by optimizing an RSSI loss function [46].

Zhang and Saad investigated the location of IoT devices in millimeter-wave networks. They proposed using the multipath channel state information (CSI) received by different base stations to estimate 3D localization using a convolutional autoencoder model. They combined unsupervised and supervised learning methods. They began by capturing the relative location of target devices and creating an autoencoder-based channel chart (unsupervised). The charting model was extended to a semisupervised framework, in which positioning accuracy improved by using the labeled CSI dataset with associated location information [47].

Ferreras and Talampas used the RSS to localize Lora-based fingerprinting. To improve the localization performance, they proposed using the signal strength difference (SSD), gateway information, and time difference of arrival (TDoA). They employed random forest (RF) and multilayer perceptron (MLP) machine learning algorithms [48].

Raghav et al. investigated the big-data-related IoT and proposed a localization scheme with an optimization approach by developing an enriched swarm intelligence algorithm based on an artificial bee colony that employed the extended Kalman filter (EKF) data blend technique for improving localization in the IoT for smart cities [49].

Shurrab et al. present an active sensor selection mechanism for target localization, in conjunction with a data-driven Q-learning approach (reinforcement learning). They proposed a dynamic approach to active node selection in which the trained RL agent was deployed in the first phase to select an appropriate grid. In the second phase, a selection mechanism was used to select the best nodes for that grid based on their attributes, such as location, cost, residual energy, and node confidence, with the goal of locating an unknown source [50].

For the social IoT, Zhou et al. proposed a fuzzy rough set theory and the ridge-regression extreme learning machine (RRELM) localization approach. Instead of the RSS, they first built a location fingerprint database that stored the minimum hop counts between the reference node (RN) and the anchor node (AN). They employed fuzzy rough set theory to calculate the significant degree of each AN and to eliminate insignificant ANs [51].

Anjum et al. investigated low-power wide-area networks (LPWANs) in the IoT industrial and research communities in relation to RSSI-based fingerprinting localization. They developed an accurate RSSI-to-distance mapping using deep learning techniques, followed by an analytically optimal model as the underlying ranging function for trilateration-based deterministic positioning [52].

Manasreh et al. proposed a method for optimizing the localization of an indoor Bluetooth Low Energy positioning system with a low beacon density. Their proposed method used genetic fuzzy systems (GFSs) by combining machine learning concepts. They employed the proposed methods to localize smartphones by utilizing RSSI values from 13 beacons [53].

Aqeel et al. investigated the effects of LoRaWAN and communication channels on node localization, as well as RSSI-based node localization in a sandstorm environment. They used machine learning algorithms, such as support vector regression and gaussian process regression, to generate a unique signature for each location. They fed RSSI features into machine learning models as input location fingerprints [54].

Panduman et al. presented Smart Environmental Monitoring and Analytical in Real-Time (SEMAR), an IoT server platform. The SEMAR platform made use of an API to service various IoT applications, including localization [55].

Chen et al. investigated the significance of localization in the IoT as well as the impact of WiFi propagation characteristics that were sensitive to the human body. These characteristics were used to create location fingerprints. To deal with the dynamic environment, they proposed Fidora, a WiFi-based localization system based on domain adaptation with a cluster assumption. According to the authors, Fidora localized different users using labeled data from only a few people and localized the same user in different environments without labeling any new data [56].

Wu et al. proposed a system architecture for spatial–temporal traceability that made use of IoT and digital-twin technologies. They used a long-short-term-memory-network-enabled genetic indoor-tracking algorithm (GITA) for BLE localization, with ultrawideband technology used for sample labeling during the training stage. To deal with signal multipath fading and streamline the learning process, they employed a feature selection method based on the RSSI [57].

The authors investigated voice-activated AI technology and proposed a deep-neural-network-based real-time sound source localization (SSL) model for low-power IoT devices. The authors used multichannel acoustic data to parallelize convolutional neural network layers in the form of multiple streams in order to capture unique delay patterns in the low-, mid-, and high-frequency ranges and estimate the fine and coarse location of voices [58].

Ngamakeur et al. proposed a deep CNN-LSTM architecture for PIR-based indoor location estimation using deep learning. The CNN network extracted features from the PIR analog output, and the LSTM network learned temporal dependencies between the extracted features in their proposed method [59].

Based on IoT localization, Chen and Weng proposed a time-dependent visiting trip planning (TVTP) framework to find the fastest moving paths. They employed a crowd-density prediction model based on deep learning and a time-dependent visiting trip planning algorithm. To reduce prediction errors, they employed densely connected convolutional networks [60].

Jia et al. investigated the requirements of real-time IoT applications and proposed a multiagent reinforcement-learning-based distributed localization scheme (MARL). They began by recasting the localization as a stochastic game in which the goal was to maximize the sum of the negative localization errors. Each nonanchor node was then represented as an intelligent agent, with an action space that corresponded to potential locations. They employed the Q-learning framework to determine the best policy and maximize the long-term expected reward [61].

Yan et al. investigated range-free localization and proposed the LSAE algorithm, which used known network information, such as hop counts and the distances between anchor nodes, to train the stacked autoencoder (SAE) model [62].

Gang et al. investigated the relationship between underwater communication IoT-based UWSNs and the united nations’ Sustainable Development Goals (SDGs) [63].

Table 2 contains a summary of related studies. As shown in Table 2, the majority of related studies concentrate on range-based localization that employs the RSS for indoor localization. Furthermore, new algorithms, such as the generative adversarial networks (GAN) [64], are barely addressed in the literature.

As shown in Table 2, the majority of the current literature focuses on indoor localization, which employs the RSSI to calculate range.

Factor graphs are a type of machine learning technique that graphically represents probabilistic models commonly used in machine learning applications such as parameter estimation, data compression, and feature selection. In the field of IoT localization, factor graphs have been used to model the relationships between various sources of information, such as time of arrival (TOA) and angle of arrival (AOA) measurements, in order to estimate the position of IoT devices. By graphically modeling these relationships, factor graphs provide a way to efficiently capture the uncertainty and dependencies in the data, resulting in improved localization performance. In the context of IoT localization, a number of works have made significant contributions to the field of probabilistic graphical models. The authors of [65] investigated indoor localization using factor graphs. They combined ranging and fingerprinting to achieve an appealing level of accuracy. In order to improve accuracy, their proposed method increased the computational complexity while decreasing deployment costs. They assessed the effectiveness of their proposed framework for hybrid UWB and WiFi localization. Another study proposed a unified factor-graph-based framework for passive localization [66]. The authors took into account ToA measurements and dealt with uncertainties such as link failure or unknown receiver location. Xiong et al. [67] presented a statistical iterative model based on factor graphs to replace the $l_{2}$ loss with $l_{p}$ under an impulsive noise for ToA-based localization. The authors of [68] looked at simultaneous localization and mapping (SLAM) for self-driving cars and proposed a Kalman filter (factor-graph-based solution) to improve the localization accuracy.

Cooperative localization is a powerful technique for IoT localization in wireless networks. This technique involves multiple devices working together to estimate their relative positions. In cooperative localization, devices share information with one another, such as AOA measurements, to improve the accuracy of their position estimates. This is especially useful in wireless networks where a single device may have limited information about its surroundings, and thus may not be able to accurately determine its position. By combining information from multiple devices, cooperative localization can achieve much more accurate position estimates compared to individual devices working in isolation. Recent advances in cooperative localization, such as the use of parametric Bayesian methods, have shown great potential in providing convergence-guaranteed solutions in massive networks. The authors can refer to [69,70,71] for more information on this promising technique for IoT localization. Machine learning algorithms can provide sophisticated data analysis and modeling capabilities that can be used to improve a cooperative localization algorithm’s performance. Machine learning algorithms can be used to identify and mitigate the effects of various sources of error and interference in IoT networks, such as radiofrequency interference and multipath fading.

**Table 2:** Summary of the related studies reviewed in this paper [2020 to 2022]. “-” means that the feature is not clearly addressed in the paper, for instance, when it is not mentioned that it has specific application in indoor or outdoor environments.

No.	Ref.	Publication Year	Range-Based or Range-Free	Indoor or Outdoor	Measurement Tech.	Machine Learning Tech.	IoT Tech.	Description
1	[26]	2020	Range-based	Outdoor	RSS	Linear regression SVM, KNN, random forest	LoRaWAN	Benchmark for measurements
2	[28]	2020	Range-based	Indoor	RSS	ANN, Levenberg–Marquardt, Bayesian regularization, backpropagation	-	
3	[29]	2020	-	-	-	CNN	NB-IoT, LoRa, RFID, SigFox, ZigBee	Middleware-free platform for IoT tracking
4	[30]	2020	Range-based	Indoor	RSS	SVM, KNN, random forest	-	Outlier detection
5	[31]	2020	Range-based	Indoor	RSS	Deep learning CNN	ZigBee	-
6	[32]	2020	Range-based	Indoor	RSS	Deep neural networks	ZigBee	Single-target localization
7	[33]	2020	Range-based	-	-	Multidimensional scaling (MDS)	-	Outlier detection for non-line-of-sight localization
8	[34]	2020	Range-based	Indoor	RSS	Deep learning	5G	3D Localization
9	[35]	2020	Range-based	Indoor	RSS	ANN, KNN	BLE	Fingerprint localization
10	[36]	2021	-	Outdoor	DoA	Unsupervised NC-MUSIC	-	Vehicular network
11	[37]	2021	Range-based	Indoor	RSS	Extreme learning, fuzzy, swarm intelligence	-	Hybrid tracking algorithm
12	[38]	2021	Range-based	Indoor	RSS	Deep reinforcement learning (Q-learning)	-	Markov decision process
13	[39]	2021	Range-based	Indoor	RSS	Deep neural network	-	Fingerprint localization
14	[40]	2021	Range-based	Outdoor	RSS	Semisupervised transfer learning	LoRaWAN	RSS besides signal-to-noise ratio and timestamps
15	[41]	2021	Range-based	-	-	Deep learning	-	Low-rank matrix formation
16	[42]	2021	Range-based	Indoor	RSS	Random forest	-	Smart building
17	[43]	2021	Range-based	Indoor	RSS	Random forest, ANN, decision tree, SVM, KNN, deep learning, linear regression	BLE	-
18	[44]	2021	Range-based	Indoor	RSS	Deep learning	-	Fingerprint localization
19	[45]	2021	Range-based	Indoor	RSS	Random forest, KNN, SVM, decision tree	BLE	Fingerprint localization
20	[46]	2021	Range-based	Outdoor	RSS	Deep learning	6G	UAVs
21	[47]	2021	Range-based	Indoor	CSI	Autoencoder	-	Millimeter- wave
22	[48]	2021	Range-based	Outdoor	RSS	Random forest, multilayer perceptron	LoRa	Using signal strength difference
23	[49]	2022	Range-based	Indoor	CSI	Swarm intelligence, extended Kalman filter	-	Big data, Smart Cities
24	[50]	2022	-	Outdoor	-	Reinforcement learning (Q-learning)	-	Grid Network
25	[51]	2022	Range-free	-	Hopping	Ridge-regression extreme learning	-	-
26	[52]	2022	Range-based	Outdoor	RSS	Deep learning	LPWAN	Fingerprint localization
27	[53]	2022	Range-based	Indoor	RSS	Genetic fuzzy systems	BLE	-
28	[54]	2022	Range-based	Outdoor	RSS	Support vector regression, gaussian process regression	LoRaWAN	Fingerprint localization
29	[56]	2022	Range-based	Indoor	RSS/CSI	Clustering using Fidora	-	Transfer learning method
30	[57]	2022	Range-based	Indoor	RSS	LSTM	BLE, UWB	Tracking
31	[58]	2022	-	Indoor	Sound	Deep neural network, CNN	-	-
32	[59]	2022	-	Indoor	PIR	CNN-LSTM	-	Using analog signals
33	[60]	2022	Range-based	Outdoor	-	Crowd density, deep learning, time-independent visiting trip, CNN	-	-
34	[61]	2022	Range-based	Indoor	RSS	Multiagent reinforcement learning	-	Distributed localization
35	[62]	2022	Range-free	-	Hopping	Autoencoders	-	-

**Figure 3:**
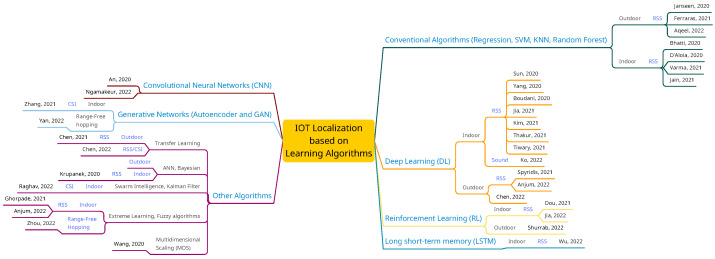
Classification of the reviewed literature, with emphasis on the learning algorithms used [28,29,31,32,33,34,37,38,39,40,41,43,44,46,47,49,50,51,52,56,57,58,59,60,61,62].

Figure 3 further categorizes the literature based on the learning algorithms used. As shown in the graph, deep learning and traditional methods such as regression, SVM, and KNN have attracted more researchers, whereas other learning algorithms such as generative networks are not adequately addressed in the literature.

## 3. Applications of Learning in IoT Localization

This section discusses the use of machine learning (ML) algorithms in IoT localization. There are some well-known IoT localization applications. Asset tracking is one of the most common applications of IoT localization. For example, a company may use IoT devices to track the movement and location of its vehicle fleet or commodity inventory [72]. This can help the company improve the effectiveness of its field service operations as well as streamline its supply chain and logistics procedures. Another industry that benefits from IoT localization is public safety [73]. Emergency responders, for example, can use IoT devices to track and locate first responders in real time to better coordinate their efforts and ensure everyone’s safety [74]. The localization of IoT devices is also applicable in smart city and smart home settings [75]. A smart home system, for example, could use IoT localization to monitor occupants’ movements throughout the house and make necessary adjustments to lighting, temperature, and other settings. The movement of residents and visitors can also be tracked using IoT localization, allowing a smart city to maximize resource utilization and improve the overall quality of life for its residents.

Learning algorithms can be used in several ways to improve the accuracy and reliability of IoT localization systems. Some of these applications are summarized as follows:

- **Calibration**: Learning algorithms can be used to calibrate sensors and other IoT device components, ensuring that they function correctly and provide accurate data. This can help to improve the overall accuracy of the location estimates [76].
- **Noise reduction**: Machine learning algorithms can be used to remove noise and other sources of error from data from IoT devices. This can aid in improving the accuracy of location estimates by reducing the impact of errors and other factors that can distort the data. Machine learning algorithms can be used to identify the most relevant features in data collected from IoT devices, thereby improving location estimation accuracy by focusing on the most important factors [18].
- **Model selection**: machine learning algorithms can be used to identify the best model or combination of models for a given application, improving location estimation accuracy by selecting the best model that fits the data.
- **Improving accuracy**: large quantities of data can be analyzed by machine learning algorithms to provide more accurate estimates of device location.
- **Automating the localization process**: machine learning has the potential to automate the process of determining device location, removing the need for manual input.
- **Adapting to changes in the environment**: to provide more accurate location estimates, machine learning algorithms can adapt to changes in the environment, such as new obstacles or changes in signal strength.

## 4. Challenges of Learning-Based IoT Localization

Several challenges must be overcome before learning algorithms for IoT localization can be used effectively. One challenge is the need for a large quantity of high-quality data to train the algorithms. Another issue is the computational complexity of deep learning algorithms, which can necessitate a significant investment in training and implementation. Finally, there are concerns about the interpretability of deep learning models because it may be difficult to understand how they arrived at their predictions. An IoT device, for example, could be trained to move in a specific way to improve the quality of the data it collects (for instance, by following a given path or turning in a specific direction). Data collection that yields precise location estimates may result in rewards for the device, whereas inaccurate estimates may result in penalties. The device would gradually learn how to change its behavior in order to maximize its rewards. One potential issue with using reinforcement learning for IoT localization is the need for a well-defined incentive signal that accurately reflects the location prediction accuracy. Furthermore, it may be necessary to carefully balance the demands of gathering high-quality data and protecting device resources (such as battery life).

- **Data quality**: The quality of data collected from IoT devices heavily influences the accuracy and dependability of location estimates. If the data are noisy, incomplete, or corrupted, the learning algorithms may struggle to locate the devices accurately.
- **Computational complexity**: Deep learning algorithms, for example, can require significant computational resources to train and deploy. Because IoT devices may have limited processing power and storage capacity, this can be difficult.
- **Limited data**: In some cases, the data collected by IoT devices may be limited in quantity or quality, making it difficult to train accurate learning models. This is especially challenging in applications where devices are deployed in unusual or rare environments, or where data are highly variable.
- **Concerns about privacy**: The use of learning algorithms in IoT applications can raise concerns about the privacy of device data. The need for accurate location estimates must be carefully balanced in some cases with the need to protect users’ privacy.
- **Security risks**: Because learning algorithms are vulnerable to hacking and other forms of tampering, their use in IoT applications can pose security risks. Appropriate security measures must be implemented to protect the data and the integrity of the learning algorithms.
- **Model selection**: Choosing the best machine learning algorithm for a particular localization task can be difficult. Different algorithms have different strengths and weaknesses, making it difficult to select the best one for the job.
- **Model interpretability**: Deep learning neural networks, for example, are notoriously difficult to interpret. This can make it difficult to understand why a particular prediction was made and to improve the model.

There are numerous challenges, but there are also numerous solutions. To improve the quality of the training data, techniques such as data cleaning and normalization can be used. These methods are commonly known as preprocessing techniques. For example, model compression can be used to reduce the computational resources needed to run machine learning algorithms. Different machine learning algorithms can be compared and evaluated using metrics such as accuracy and the computational resources required to choose the best machine learning algorithm for a specific task. To improve interpretability, techniques such as feature importance analysis and model visualization can be used. Transfer learning techniques can also be used to leverage pretrained models on large datasets, allowing knowledge to be transferred to smaller datasets. Federated learning can also be used to train models while maintaining data privacy because the data are kept on the IoT devices and only model parameters are exchanged. Furthermore, data augmentation techniques such as random cropping, rotation, or translation can be used to generate synthetic data to address the limited quantity of data. Unsupervised learning techniques such as clustering can be used in some cases to identify patterns in data where labels are not available. Edge computing can also be used to reduce the quantity of data that must be transmitted to the cloud, saving network bandwidth and reducing the computational burden on the cloud. Encryption and secure communication protocols can be used to protect device data and learning algorithms, addressing privacy and security concerns. Machine learning techniques that preserve privacy, such as differential privacy, can be used to provide a mathematically rigorous way of ensuring the privacy of device data. To summarize, we can overcome these challenges and develop accurate and reliable machine learning models for IoT localization by carefully considering the limitations of machine learning and IoT devices and leveraging preprocessing techniques, transfer learning, federated learning, data augmentation, unsupervised learning, and privacy-preserving techniques.

We also recognize that the evidence has limitations that must be taken into account when interpreting the findings. In light of the fact that our review lacked a formal method for assessing bias, the risk of bias across the included studies is a concern.

## 5. Lesson Learned and Future Trends

### 5.1. Lesson Learned

Reviewing the current literature reveals that, while there are numerous studies on Learning-based IoT localization, there are still many unresolved issues. For example, the majority of current research focuses on range-based localization, which employs basic learning algorithms such as KNN, SVM, decision tree, and random forest. A few are concerned with autoencoders and reinforcement learning. The authors developed a model for fingerprint localization in both indoor and outdoor environments. Range-based models, particularly those built on the RSS, are sensitive to environmental changes. One solution is to employ transfer learning techniques such as few-shot learning. Another important aspect that was also addressed in the challenges of learning-based IoT localization is the lack of data or data of poor quality. One method addressed in the literature by “outlier detection” focused on locating the sources of erroneous data that increased localization errors. Learning algorithms could be used as promising solutions to this problem. It should also be noted that some practical constraints, such as battery life, are frequently overlooked when addressing IoT localization.

Given these limitations, readers are advised to exercise caution when interpreting the findings and consider future research that addresses these shortcomings, such as employing a formal risk of bias assessment or conducting more rigorous, standardized studies to improve the quality of evidence. We state that this survey paper is not registered with any registry.

### 5.2. Future Trends

Making better decisions and streamlining processes is made possible by the use of learning algorithms in IoT localization, which can improve the accuracy and dependability of location estimations. It is expected that the use of learning and machine learning in IoT localization will expand and change in the coming years. The following are some potential future trends:

- Machine learning algorithms are likely to become more integrated into the hardware and software components of IoT devices, allowing them to process and analyze data in real time and adapt to changing conditions.
- Real-time location tracking: machine learning algorithms will continue to improve the speed and accuracy of real-time location tracking, allowing devices to be tracked in real time with low latency.
- Context-aware localization: to provide more accurate location estimates, machine learning algorithms will consider contextual information such as the device’s surroundings and environment.
- More sophisticated learning algorithms: as machine learning techniques advance, more sophisticated algorithms capable of handling larger and more complex datasets will most likely be developed, resulting in more accurate location estimates.
- Increased use of machine learning in edge computing: Edge computing, which involves processing data at the network’s edge rather than in the cloud, is becoming more important in IoT applications. In the coming years, machine learning algorithms that can run efficiently on edge devices will be in high demand.
- Machine learning applications are expanding into new areas. Machine learning techniques are likely to be applied to a broader range of IoT applications as they advance, including transportation, healthcare, and environmental monitoring.

## 6. Conclusions

This paper provided a thorough overview of the application of machine learning algorithms in IoT localization systems. We reviewed the existing literature released between 2020 and 2022 to understand how learning algorithms were used to solve IoT localization problems, as well as the current challenges associated with these systems. According to the findings of this review, machine learning algorithms are increasingly being used to improve the accuracy and reliability of IoT localization systems, with applications ranging from real-time location tracking to context-aware localization. However, there are some drawbacks to using machine learning algorithms in IoT localization, such as data quality and quantity, computational resources, and model selection. Various techniques, such as data preprocessing, model compression, and model interpretability, can be used to overcome these challenges. According to the paper, machine learning algorithms have the potential to revolutionize the field of IoT localization, and the future of this field appears bright.

## Figures and Tables

**Table 1:** A summary of existing surveys, organized by type of localization, year range, focus, and machine learning techniques covered.

Reference	Type of Localization	Year Range	Focus	Machine Learning Techniques
Asaad and Maghdid [9]	Outdoor and indoor	2019–2021	Classification based on wireless techniques, sensors, environments, objects, and metrics	-
Moradbeikie et al. [16]	GNSS-free outdoor localization for the IoT	2018–August 2021	Cloud, fog, and edge-AI-based architectures	Mentions some deep learning in a limited number of related works
Kordi et al. [17]	Wireless emerging IoT indoor localization	2016–2019	Classification based on localization techniques	Very limited number of learning-based methods (papers) are covered
Rathin Chandra Shit et al. [5]	IoT localization	Before 2018	Taxonomy of localization methods (self-determining, training-dependent)	Focuses on localization (not learning); Covers limited papers on fingerprinting, stochastic model process, machine learning approaches
Li et al. [18]	Location-enabled IoT	Before 2020	Positioning techniques, error sources, error mitigation	Focus on error source and error mitigation, limited learning-based methods are introduced
Atitallah et al. [19]	Deep learning and big data analytics for smart cities	2017–2018	Learning-based localization in passing	Addresses only two learning-based publications
Bellavista-Parent, Torres-Sospedra, and Pérez-Navarro [20]	WiFi-based indoor positioning	2016–2021	Focuses only on WiFi-indoor localization	Categorization based on machine learning techniques (DRL, ELM, CNN, DNN, BPNN, CapsNet, SDA, VAE, DQN, SVM, Bayesian methods)
Alam, Faulkner, and Parr [21]	Non-RF techniques for unobtrusive indoor positioning	2010–2020	Location-based services using visible light, infrared, vibration, pressure, and an electric field	-
Khan et al. [22]	Location-aware IoT	Before 2021	Classification based on applications, smartphone usage, security, energy efficiency, target recovery, target prediction capabilities	-
Farahsari et al. [23]	Indoor positioning systems for IoT-based applications	Before 2022	Classification based on scale, environment, and initial user, communication technologies (short-range, long-range, signal-type)	-
Mahmood et al. [24]	Machine learning algorithm applications for future IoT	Before 2022	Evaluation of future communication networks (3G to 6G), algorithm type (heuristic, supervised, unsupervised, deep learning, reinforcement learning, deep reinforcement learning, federated learning)	Focuses only on communication networks (3G to 6G) based on machine learning algorithms
Alhomayani and Mahoor [25]	Fingerprint-based indoor positioning	Before 2020	Fingerprint-based indoor positioning	Only deep learning methods are covered
**Our Survey**	Indoor, outdoor, fingerprint, LoRaWan,	2020 to the end of 2022	Localization techniques with machine learning support	All the machine learning techniques in the literature

## Data Availability

Not applicable.

## Abbreviations

The following abbreviations are used in this manuscript:

DNN	Deep neural network
DL	Deep learning
IoT	Internet of things
LSTM	Long-short term memory
SVM	Support vector machines
BLE	Bluetooth Low Energy
MLP	Multilayer perceptron
KNN	k-Nearest neighbors
ANN	Artificial neural networks
UWB	Ultrawideband
RFID	Radiofrequency identification
CNN	Convolutional neural network
MDS	Multidimensional scaling
EDM	Euclidean distance matrix
ELM	Extreme learning machine
MDP	Markov decision process
AE	Autoencoder
SSD	Signal strength difference
TDoA	Time difference of arrival
EKF	Extended Kalman filter
